# Traditional alcoholic beverages and their value in the local culture of the Alta Valle del Reno, a mountain borderland between Tuscany and Emilia-Romagna (Italy)

**DOI:** 10.1186/s13002-016-0099-6

**Published:** 2016-06-22

**Authors:** Teresa Egea, Maria Adele Signorini, Luca Ongaro, Diego Rivera, Concepción Obón de Castro, Piero Bruschi

**Affiliations:** Dipartimento Biología Aplicada, Escuela Politécnica Superior, Universidad Miguel Hernández, Ctra. Beniel Km 3.2, 03312 Orihuela, Alicante Spain; Dipartimento di Scienze delle Produzioni Agroalimentari e dell’Ambiente, Università degli Studi di Firenze, Florence, Italy; Dipartimento di Biologia, Università degli Studi di Firenze, Florence, Italy; Agenzia Italiana per la Cooperazione allo Sviluppo - Italian Development Cooperation Agency, Florence, Italy; Dipartimento Biología Vegetal, Campus de Espinardo, Universidad de Murcia, Murcia, Spain

**Keywords:** Liquors, Fermented beverages, Distillates, Aromatized wines, Ethnobotany

## Abstract

**Background:**

Traditional alcoholic beverages (TABs) have only received marginal attention from researchers and ethnobotanists so far, especially in Italy. This work is focused on plant-based TABs in the Alta Valle del Reno, a mountainous area on the border between Tuscany and Emilia-Romagna regions. The aims of our study were to document local knowledge about TABs and to analyze and discuss the distribution of related knowledge within the investigated communities.

**Methods:**

Field data were collected through semi-structured interviews. The relative importance of each plant species used to prepare TABs was assessed by calculating a general Use Value Index (*UV*_general_), a current *UV* (*UV*_current_) and a past *UV* (*UV*_past_). We also assessed personal experience of use by calculating effective and potential *UV* (*UV*_effective,_*UV*_potential_). A multivariate analysis was performed to compare ingredients in recipes recorded in the Alta Valle del Reno with those reported for neighboring areas.

**Results:**

Forty-six plant species, belonging to 20 families, were recorded. Rosaceae was the most significant family (98 citations, 19 species), followed by Rutaceae (15, 3) and Lamiaceae (12, 4). The most important species was *Prunus cerasus* L. (*UV*_general_ = 0.44), followed by *Juglans regia* L. (0.38), *Rubus idaeus* L. (0.27) and *Prunus spinosa* L. (0.22). Species with the highest *UV*_current_ were *Juglans regia* (0.254), *Prunus cerasus* (0.238) and *Citrus limon* L. (0.159). The highest *UV*_effective_ values were obtained by *Prunus cerasus* (0.413), *Juglans regia* (0.254), *Rubus idaeus* (0.222) and *Citrus limon* (0.206). We also discuss the results of the multivariate analysis.

**Conclusions:**

TABs proved to occupy an important place in the traditional culture and social life of the studied communities. Moreover, data highlight the local specificity and richness of this kind of tradition in the Alta Valle del Reno, compared to other Italian areas. Some plant ingredients used for TABs have potential nutraceutical and even therapeutic properties that are well known by local people. These properties could constitute an additional economic value for TABs' commercialization, which in turn could promote the local rural economy.

## Background

Alcoholic beverages have been a distinctive component of many cultures for thousands of years [1, 2] and in most human communities they are still part of the traditional knowledge here intended in the sense of Dutfield [3]. As it is commonly well known, human consumption of fermented products is documented in texts dating back to the 2^nd^ millennium BC in West Asia [4, 5]. Among the most common alcoholic beverages in European and the Mediterranean area, wine is cited in the Bible and in ancient Greek and Latin literature, beer was already known in ancient Egypt, and cider is probably as old as the other two cited beverages. In human communities where alcohol was traditionally consumed, production of alcoholic beverages commonly occurred on a small scale as a household or artisanal activity, particularly where - or when - agricultural surpluses were available [6]. In many cultures, drinking alcohol was an occasional activity shared by people within the communities, often associated with festivals or other special occasions [6]. In many places around the world, traces of these traditional customs originating from tribal and village societies still persist [7–11].

Traditional alcoholic beverages (TABs) are homemade and informal preparations produced at local or family level. The World Health Organization [6] includes these traditional drinks in the so-called “unrecorded alcohol,” highlighting its cultural, social and economic importance around the world. It has been estimated that almost one-quarter (24.8 %) of all the alcohol consumed worldwide is drunk in the form of unrecorded alcohol [6]. In some countries, particularly in southeastern Asia and in the eastern Mediterranean region, unrecorded alcohol consumption represents more than 50 % of total alcohol consumption [6]. A wide variety of TABs can be found in different parts of the world, and often the social context in which these beverages are produced and consumed is also of interest [6]. Yet, until now such beverages have only received marginal attention by ethnobotanists. Some studies concern traditional fermented beverages and food in Africa [11–19], Asia [19–21] and Central-South America [19, 22, 23], while information on TABs is relatively scarce for European countries, with only a few recent exceptions. Among these, a paper on juniper beer in Poland [24], one on the traditional Greek fruit distillate *Koumaro* [25], some studies on the preparation of the traditional French *liqueur de cassis* [26] and further references here reported, a review of traditional fermented plant food and beverages in Eastern Europe [27] and a general review on the use of aromatic plants in alcoholic beverages [28]. In Italy, several popular booklets or websites are available on the subject, see for instance [29–34], but specific scientific investigations are extremely scarce. Some specific studies dealing in particular with the lemon-based liquor *limoncello* and its constituents have been carried out in recent years [35–39]. Egea et al. [2] discuss the relations between traditional medicinal liquors prepared in different regions of North-Central Italy (including our study area) and neighboring European regions, together with possible links to liquors reported in ancient herbals and pharmacopeias. Several ethnobotanical studies concerning the Italian territory mention traditional home-made liquors, but usually they do not provide any further detail, such as a list of ingredients, way of preparation, social context of production and/or consumption [40–44]. It can be said that ethnobotanical field investigations or reviews especially devoted to TABs and their social, cultural and possible nutraceutical value are substantially lacking in Italy.

The present work focuses on homemade plant-based alcoholic beverages in some communities located in the Alta Valle del Reno (upper Reno valley, Italy), a mountainous area lying in the Appennino Tosco-Emiliano (Northern Apennines) on the border between the Tuscany and Emilia-Romagna regions. Our study concerns plant ingredients, preparations, different aspects of used plants including their geographical origin, and the cultural context of preparation and consumption of TABs. This study is part of a wider research project on the ethnobotany of the Alta Valle del Reno, aimed at documenting and preserving the local ethnobotanical knowledge, making it available for further scientific research, and highlighting its potential applications for local economic and environmental developments.

The specific aims of the present work are:

(i) to document the use of TABs and related knowledge in the Alta Valle del Reno and its possible specificity compared to the rest of Italy;
(ii) to analyze distribution and other aspects of this knowledge in the area (e.g., whether or not they are still practiced, if the knowledge is gendered, and others);
(iii) to discuss potential factors affecting patterns of distribution of TABs knowledge.

## Methods

### Study area

**Fig. 1:**
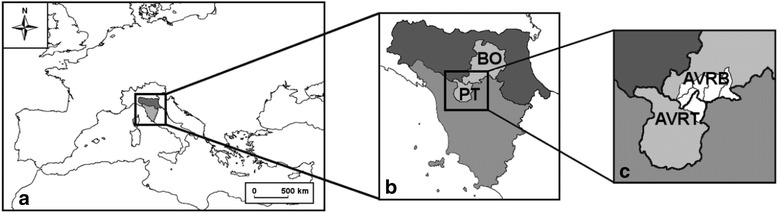
Study area. **a** Position of Tuscany (grey) and Emilia-Romagna (dark grey) in Europe **b** PT: province of Pistoia (Tuscany); BO: province of Bologna (Emilia-Romagna). **c** In white: the six municipalities involved in the study; AVRT: the Alta Valle del Reno, Tuscan side; AVRB: the Alta Valle del Reno, Emilian side

The communities under study are located in a mountainous area crossed by the river Reno (Fig. 1). These communities are scattered in six municipalities: Sambuca Pistoiese (Tuscany), Castel di Casio, Camugnano, Castiglione dei Pepoli, Granaglione and Porretta Terme (Emilia-Romagna) (Fig. 1). The population of the whole study area amounts to around 20,300 inhabitants (data obtained from the municipalities’ administrations). The territory—covering about 360 km^2^, nearly 10 % of which falls within the regional park of Suviana and Brasimone lakes—lies between northern Tuscany and southern Emilia-Romagna, limited to the north by the Bologna plain (Emilia-Romagna) and to the south by the Pistoia plain (Tuscany).

The Alta Valle del Reno can be considered a borderland between northern and southern Italy under different aspects. From a biogeographical point of view, it lies along the line dividing the Middle-European region from the Mediterranean one [45, 46]. Historically, this natural barrier has been, through the centuries, a site of exchange, war and refuge, separating different territorial authorities or domination [47–49]. For example, there are Celtic and Etruscan settlements, the area was dominated by the Longobardi and the Byzantine exarchate of Ravenna, and then by the Grand-Duchy of Tuscany and the Papal States. Finally, during the Second World War, this part of the Apennines was the location of a crucial section of the Gothic Line. The Alta Valle del Reno is also crossed by the pilgrim road “via Francigena” that runs from central Europe to Rome, an important connecting route between northern and southern Europe since the 6^th^ century.

The Alta Valle del Reno represents at the same time a transit place, a separation, and a meeting land that houses a unique identity. This area is distinguished even by a peculiar language system, called Gallo-Tuscan [47, 50]. This “border dialect” [51, 52] is spoken only along one of the most important dialect barriers in Italy, the so-called “La Spezia-Rimini line,” which separates eastern Romance languages from western ones [50].

The Alta Valle del Reno has also peculiar geomorphological, climatic and vegetation features. From a geomorphological point of view, the study area is characterized by a hilly to steep mountainous morphology, with elevations ranging from 271 m a.s.l. at Ponte di Verzuno (Camugnano) to 1430 m a.s.l. at Passo del Termine (Granaglione). The climate is temperate-subcontinental, characterized by cold and snowy winters alternating with rather hot summers. The average annual precipitation reaches 2000 mm in some inland localities (the Italian average value is around 760 mm) [53, 54]. The area is mainly covered by broadleaved woodlands: chestnut woods (*Castanea sativa* Miller) and mixed woods dominated by deciduous oaks (*Quercus pubescens* Willd. and *Quercus cerris* L.) are replaced at higher altitudes by beech forests (*Fagus sylvatica* L.). Conifer reforestations are also frequent, as well as scattered meadows and pastures. Small agricultural plots along with abandoned fields gradually reconquered by natural vegetation complete the landscape.

This peculiar geographical, historical and environmental context originated not only an extraordinary cultural wealth but also a high biological diversity. For this reason, in the area, there are several Sites of Community Importance (SCI) issued by the European Community and Rete Natura 2000 (92/43/CEE). Up to the 20^th^ century, the rural economy of the study area was based on agriculture, livestock farming, forestry and hunting. The cultivation of chestnut trees has been the most important agricultural activity for centuries, followed by cereals and forage; home gardens and vineyards are also widespread—the latter holding a more social than economic importance in local communities [55]. Forest exploitation for charcoal, timber and non-timber products was essential for the household economy up to about 40 years ago.

The geographical and historical isolation of the valley contributed creating a close relationship between people and the surrounding environment, resulting in a rich traditional knowledge about local plants and their uses. However, hardships and difficulties linked to the geomorphology, climate and relative isolation of the valley, combined with the socio-economical changes occurring in many Italian rural areas, have led to a significant depopulation since the beginning of the 20th century. Along with a declining rural population, many traditional lifestyles and activities are also disappearing, as well as the related knowledge.

### Data collection

Field data on TABs were collected from May 2009 to March 2015 through interviews with local informants. These interviews were conducted in the above-mentioned villages located on both sides of the Reno river (Fig. 1. Tuscany area: AVRT; Emilia-Romagna area: AVRB). After preliminarily introducing the research and its purpose in each municipality, informants were contacted through “snowball” sampling, i.e., asking an informant to suggest other informants [56]. Only persons who were born in the study area or had been living there for a long time and consequently had direct experience of local habits were taken into consideration as informants. We also made sure that their knowledge of TABs was acquired from traditional culture only, not from books, magazines or other media. Information was mainly collected through semi-structured interviews, sometimes carried out with the help of informants’ relatives or friends acting as intermediaries. Such mediators were of great importance in creating a familiar and friendly environment during the interviews. Intermediaries’ help was also crucial in carrying out the interviews at least partially in the local dialect and in stimulating the informants’ memory about traditional uses of plants, anecdotes or common experiences. As usually recommended in ethnobotanical investigations (see among the others [57]), interviews were carried out as general friendly conversations, in which the informant’s attention to the plant uses was recalled by the interviewer from time to time. During the interview, personal and socio-economic features of each informant (gender, age, education rate, occupation etc.) were also recorded. Interviews were carried out complying with the ethics guidelines commonly followed in ethnobotanical studies [58], and the informants’ consent was obtained prior to the interviews. The total number of informants who mentioned plant-based TABs was 63. Information was also obtained through participant observation by one of us (TE) who had spent long periods living in the villages, sharing the everyday routine with the informants and taking part in the traditional practices of the communities.

Collected data concern preparation and consumption of any kind of plant-based homemade alcoholic beverage and include information on:

- Plants: local name/s; used plant part; if the plant is wild or cultivated in the area; possible further notes.
- Uses: type of TABs (see below); description of how the beverage is prepared (with dosage of each ingredient if possible); preservation and consumption (including social context and possible related customs and rituals); whether the use is still practiced or not; direct or indirect experience of the use by the informant.
- Medical/nutraceutical properties of TABs according to local traditional knowledge (including local names of diseases).

TABs prepared and consumed by rural communities living in the studied area were classified into the following categories.

- “Distillates,” made by distilling with alembics different sorts of fermented fruits.
- “Liquors,” consisting of macerations in grappa or in 95 % ethanol of different plant parts (fruits, seeds, roots, leaves or flowers), mixed with a syrup made with sugar and water.
- “Fermented beverages,” low-alcohol, short-lasting beverages made from fermented fruits or flowers, sometimes with the addition of sugar and/or water.
- “Aromatized wines,” obtained by aromatizing red wine with local or exotic aromatic plants (spices), either by maceration or heating (mulled wine). These can be served hot or warm, and alcohol content is widely variable.

Both wild and cultivated plants quoted by people as being used to prepare TABs were recorded. We excluded only those species that are primarily cultivated to serve as a base for the alcoholic beverage or as sweeteners, e.g., *Vitis vinifera* L. (wine, alcohol), *Hordeum vulgare* L. and *Humulus lupulus* L. (beer), and *Beta vulgaris* L. (sugar for liquors and fermented beverages).

### Plant identification

Samples of the plants used for TABs were gathered with the informants when possible (in some cases, weather conditions, age or health conditions of the informant did not allow for this activity). Common cultivated plants (e.g., *Rosmarinus officinalis* L., *Citrus limon*) and exotic spices (e.g., *Cinnamomum verum* J.Presl., *Syzygium aromaticum* (L.) Merr. & L.M.Perry) were not collected. Collected plants were exsiccated, mounted as herbarium specimens following standard procedures and identified using the *Flora d´Italia* [59]. Voucher specimens were deposited in the herbarium FIAF (University of Florence).

Plant nomenclature (Table 1) is in accordance with The Plant List [60]. In a few instances (e.g.: *Mentha spicata* L.), we adopted the botanical species name in a broad sense (species *sensu lato*), as it is more suitable when dealing with ethnobotanical data. We collated information on the life form, general geographical distribution (chorology) and wild/cultivated status for each identified plant species using the Flora d'Italia [59]. From these data, we elaborated the biological spectrum and the chorological spectrum (i.e., the relative number of species belonging to each life form or chorological type, expressed as a percentage of the total).

**Table 1:** Plants used in the preparation of Traditional Alcoholic Beverage (TABs) in Alta valle del Reno

Scientific name	Botanical family	Local names	Wild/ Cultivated	Informants mentioning the plant	Citations	Uses (Used parts)	*UV*				
							_*general*_	_*current*_	_*past*_	_*effective*_	_*potential*_
*Aloysia citriodora* Palau	Verbenaceae	Pl: acedrina, cedrina	C	3	3	Liq (Lv)	0.048	0.032	0.016	0.032	0.016
*Camellia sinensis* (L.) Kuntze	Theaceae	Pl: té	A	2	2	Liq (Lv)	0.032	0.032	0.000	0.032	0.000
*Castanea sativa* Miller	Fagaceae	Fr: castagne	W	1	1	Dist (Fr)	0.016	0.000	0.016	0.000	0.016
*Cinnamomum verum* J. Presl	Lauraceae	Pl: cannella	A	6	8	Liq (Bk) Arom (Bk)	0.127	0.111	0.016	0.127	0.000
*Citrus limon* (L.) Burm. f.	Rutaceae	Pl: Fr: limone	C	11	13	Liq (Fr Lv) Arom (Fr)	0.206	0.159	0.048	0.206	0.016
*Citrus sinensis* L. Osbeck	Rutaceae	Pl: Fr: arancio	C	1	1	Arom (Fr)	0.016	0.000	0.016	0.016	0.000
*Coffea arabica* L.	Rubiaceae	Pl: caffé	A	1	1	Liq (Fr)	0.016	0.000	0.016	0.016	0.000
*Cornus mas* L.	Cornaceae	Fr: corniole	W	10	10	Liq (Fr) Arom (Fr)	0.159	0.079	0.079	0.127	0.032
*Fragaria vesca* L.	Rosaceae	Fr: fragole	W	3	3	Ferm (Fr)	0.048	0.000	0.048	0.048	0.000
*Gentiana* cfr. *asclepiadea* L.	Gentianaceae	Pl: genziana	W	3	3	Liq (Uo) Arom (Uo)	0.048	0.032	0.016	0.032	0.016
*Gentianopsis ciliata* (L.) Ma. (cfr.)	Gentianaceae	Pl: genzianella	W	2	2	Liq (Uo) Arom (Uo)	0.032	0.032	0.000	0.032	0.000
*Juglans regia* L.	Juglandaceae	Pl: Fr: noce	W	23	24	Liq (Fr) Arom (Fr)	0.381	0.254	0.127	0.254	0.127
*Juniperus communis* L.	Cupressaceae	Pl: zinepro, ginepro	W	6	7	Dist (Fr) Liq (Fr)	0.111	0.063	0.048	0.063	0.048
*Laurus nobilis* L.	Lauraceae	Pl: alloro	W	2	2	Liq (Lv)	0.032	0.032	0.000	0.032	0.000
*Malus domestica* Borkh.	Rosaceae	Fr: mele, mele antiche	C	2	2	Liq (Se) Arom (Fr)	0.032	0.032	0.000	0.032	0.000
*Malus sylvestris* Miller	Rosaceae	Fr: mele selvatiche	W	1	1	Liq (Se)	0.016	0.016	0.000	0.016	0.000
*Matricaria chamomilla* L.	Compositae	Pl: Camomilla	W	1	1	Liq (Fl)	0.016	0.016	0.000	0.016	0.000
*Mentha spicata* L. (s.l.)	Lamiaceae	Pl: menta selvatica	W	2	2	Liq (Lv)	0.032	0.016	0.016	0.016	0.016
*Ocimum basilicum* L.	Lamiaceae	Pl: basilico	C	5	5	Liq (Lv)	0.079	0.079	0.000	0.079	0.000
*Pinus nigra* J.F. Arnold (s.l.)	Pinaceae	Pl: pino	C	1	1	Liq (Bd)	0.016	0.016	0.000	0.016	0.000
*Prunus avium* L.	Rosaceae	Fr: ciliegie, duroni, ciliegie innestate	W	4	4	Ferm (Fr) Liq (Fr)	0.063	0.048	0.016	0.047	0.016
*Prunus cerasifera* Ehrh.	Rosaceae	Fr: prugne selvatiche	W	1	1	Dist (Fr)	0.016	0.016	0.000	0.016	0.000
*Prunus cerasus* L.	Rosaceae	Fr: amarene, marasche selvatiche, ciliegie amarene, ciliegie selvatiche	W	22	28	Ferm (Fr) Liq (Fr Lv Se) Arom (Lv)	0.444	0.238	0.206	0.413	0.031
*Prunus domestica* L.	Rosaceae	Fr: prugne	C	2	2	Dist (Fr) Ferm (Fr)	0.032	0.032	0.000	0.032	0.000
*Prunus laurocerasus* L.	Rosaceae	Pl: lauro	C	3	3	Liq (Fr)	0.048	0.016	0.032	0.016	0.032
*Prunus persica* (L.) Batsch	Rosaceae	Fr: pesca	C	1	1	Liq (Se)	0.016	0.016	0.000	0.016	0.000
*Prunus spinosa* L.	Rosaceae	Pl: spino selvatico, spino nero, prugnolo, spini, Fr: strozzaprete, strozzigo	W	13	14	Dist (Fr) Liq (Fr) Arom (Fr)	0.222	0.079	0.143	0.111	0.111
*Pyrus communis* L.	Rosaceae	Fr: pere snace, pere cestello, pere volpine	C	4	4	Ferm (Fr)	0.063	0.000	0.063	0.063	0.000
*Pyrus pyraster* Burgsd.	Rosaceae	Fr:pere selvatiche﻿	W	3	3	Ferm (Fr)	0.048	0.000	0.048	0.048	0.000
*Robinia pseudoacacia* L.	Leguminosae	Pl: acacia	W	1	1	Ferm (Fl)	0.016	0.000	0.016	0.016	0.000
*Rosa canina* L. (s. l.)	Rosaceae	Pl: rosa canina Fr: peterlenga	W	3	3	Liq (Fl Fr)	0.048	0.000	0.048	0.048	0.000
*Rosa* cv.	Rosaceae	Pl: rosa antica	C	2	2	Liq (Fl Lv)	0.032	0.000	0.032	0.032	0.000
*Rosmarinus officinalis* L.	Lamiaceae	Pl: rosmarino	W	2	2	Liq (Ap)	0.032	0.032	0.000	0.032	0.000
*Rubus hirtus* W. et K.	Rosaceae	Pl: rovo	W	1	1	Ferm (Fr)	0.016	0.000	0.016	0.016	0.000
*Rubus idaeus* L.	Rosaceae	Pl: Fr: lampone, lampone selvatico	W	15	17	Ferm (Fr) Liq (Fr)	0.270	0.048	0.222	0.222	0.048
*Rubus ulmifolius* Schott	Rosaceae	Pl: rovo di bosco, raggie Fr: more, more selvatiche,	W	6	6	Ferm (Fr) Liq (Fr)	0.095	0.032	0.063	0.095	0.000
*Rubus* cv.	Rosaceae	Fr: more domestiche	C	1	1	Ferm (Fr)	0.016	0.000	0.016	0.016	0.000
*Ruta graveolens* L.	Rutaceae	Pl: ruta	W	1	1	Liq (Ap)	0.016	0.016	0.000	0.016	0.000
*Salvia officinalis* L.	Lamiaceae	Pl: salvia	C	3	3	Liq (Lv)	0.048	0.048	0.000	0.048	0.000
*Sambucus ebulus* L.	Adoxaceae	Pl: Fr: ebbi, ebbiacci	W	1	1	Dist (Fr)	0.016	0.000	0.016	0.000	0.016
*Sambucus nigra* L.	Adoxaceae	Pl: sambuco	W	4	4	Ferm (Fr) Liq (Fr)	0.063	0.016	0.048	0.063	0.000
*Sorbus domestica* L.	Rosaceae	Fr: sorbole	W	2	2	Ferm (Fr)	0.032	0.000	0.032	0.032	0.000
*Syzygium aromaticum* (L.) Merr.& L.M.Perry	Myrtaceae	Pl: chiodi di garofano	A	8	10	Liq (Fl) Arom (Fl)	0.159	0.127	0.000	0.159	0.000
*Vaccinium myrtillus* L.	Ericaceae	Pl: Fr: mirtilli	W	11	12	Ferm (Fr) Liq (Fr)	0.190	0.079	0.111	0.175	0.015
*Vanilla planifolia* Andrews	Orchidaceae	Pl: vaniglia	A	2	2	Liq (Fr)	0.032	0.032	0.000	0.032	0.000
*Vitis labrusca* L.	Vitaceae	Fr: uva fragola	W	2	2	Liq (Fr)	0.032	0.032	0.000	0.032	0.000

Plant parts - Ap: whole aerial part; Bd: buds; Bk: bark; Fl: flowers/inflorescences; Fr: fruit; Lv: leaves; Pl: plant; Uo: underground organs; Se: seeds. Wild/Cultivated (in Tuscany and Emilia-Romagna) - A: absent; C: cultivated; W: wild (also including plants occurring both as wild and cultivated). Preparations - Arom: aromatized wines; Dist: distillates; Ferm: fermented beverages; Liq: liquors

### Bibliographic sources

We compared our data with other data extracted from bibliographic sources to assess the influence of the culture in neighboring regions. Specifically, we compared our data with studies reporting information on TABs from Emilia Romagna and Tuscany. For Emilia Romagna, we used the following sources: a collection of recipes of liquors traditionally prepared in Emilia-Romagna, available on the official website of the region [31]; a study on traditional plant-based medicine in Emilian Apennines, in the area of Parma province [61]; a survey on wild food plants traditionally consumed in the area of Bologna [62]. For the Tuscany region, instead, we used the following sources: a compendium of ethnobotanical research in Tuscany [63]; a survey on medicinal plants and food medicines in Garfagnana (Appennino Tosco-Emiliano, North-Western Tuscany), reporting several medicinal liquors [64]; a field investigation on traditional uses of plants in Firenzuola (province of Florence, Tuscany) [65].

### Data analyses and quantitative indexes

We organized the data on TABs in a simple database using Microsoft Excel. Each row (elementary record) represents a *citation*, defined as a single use reported for a single plant by a single informant [66]. We considered as distinct citations those differing from one another in at least one of the following data: species, informant and the category of use (i.e., distillates, fermented beverages, aromatized wine or liquors). Citations differing in minor aspects, such as the part of the plant used, were combined into a single citation. The number of uses was obtained by considering as distinct uses for each species those differing in category of use. In the columns of the table, the following attributes are reported for each citation: scientific plant names, botanical family, vernacular plant name/s, informant name, category of use, used plant part and all the other information concerning plant use collected in the interviews (see above “Data collection”). Tables 1, 2 and 3 synthesize data and information extracted from the primary database described above. We organized and sorted the data using the program EBtools (Signorini and Ongaro, unpubl.), a collection of scripts in Visual Basic for Applications in Microsoft Excel that performs advanced sorting, filtering, and counting of data based on specific user requirements.

**Table 2:** Informants and knowledge about TABs in Alta Valle del Reno

Informants	Number		L_s_		L_s_/T_s_		L_u_		L_u_/T_u_	
	F	M	F	M	F	M	F	M	F	M
Total	42	21	3.40 ± 2.70	2.59 ± 1.33	0.15	0.07	4.0 ± 3.69	2.63 ± 1.36	0.22	0.10
Age class										
40-60	4	1	6.25 ± 4.27	3.00 ± 0.00	0.17	0.16	6.50 ± 4.40	3.00 ± 0.00	0.17	0.14
61-80	25	14	3.44 ± 2.95	2.71 ± 1.26	0.17	0.07	4.04 ± 4.00	2.79 ± 1.31	0.31	0.10
>80	13	6	2.84 ± 1.99	2.50 ± 1.64	0.11	0.06	2.92 ± 1.93	2.50 ± 1.64	0.09	0.09
Education level										
Primary school	27	9	3.04 ± 2.74	2.89 ± 1.54	0.14	0.08	3.30 ± 3.65	2.89 ± 1.54	0.19	0.11
Middle school	9	6	4.55 ± 3.50	2.83 ± 1.33	0.14	0.09	5.11 ± 3.89	2.83 ± 1.33	0.24	0.12
High school	2	4	7.00 ± 2.83	2.25 ± 0.96	0.29	0.05	9.00 ± 1.41	2.50 ± 1.29	0.66	0.07
University degree	4	2	2.75 ± 1.26	2.00 ± 1.41	0.15	0.03	3.00 ± 1.63	2.00 ± 1.41	0.20	0.05

F = female; M = male; Ls = mean number of TAB species cited by each informant; L_u_ = mean number of TAB uses (citations) referred by each informant; L_s_/T_s_ = number of species used for TAB/total number of species cited for any ethnobotanical use by each informant; L_u_/T_u_ = number of TAB uses/total number of any ethnobotanical use cited by each informant

**Table 3:** Relevance of knowledge concerning TABs in Alta Valle del Reno (AVR) and in different Italian areas

Areas	Ethnoflora (T_s_)	Food plants (F_s_)	Plants used for TAB (L_s_)	L_s_/T_s_	L_s_/F_s_
AVR	258	129	43	0.17	0.33
Firenzuola	163	61	10	0.06	0.16
Tuscany	517	201	32	0.06	0.16
Italy	1512	580	84	0.06	0.14

T_s_: number of species reported for any ethnobotanical use; F_s_: number of species reported for any alimentary use (TAB included); L_s_: number of species used for TAB. Exotic spices used as flavors were not considered in data comparison (see the text for further explanations)

We used the Use Value index (UV) proposed by Rossato et al. [67] to assess the relative importance of each plant species used in the preparation of TABs. We calculated different types of *UV*: a General Use Value (*UV*_general_), based on citations of any TABs of that species recorded in the interviews; a Current Use Value (*UV*_current_), based only on the citations of plants reported by the informants for uses still practiced at the present time; a Past Use Value (*UV*_past_), based on the citations of plants reported by the informants as used only in the past. We also assessed personal experience of use by calculating an Effective Use Value (*UV*_effective_), based on citations of uses directly experienced by the informants, and a Potential Use Value (*UV*_potential_), based on uses known, but never practiced by them.

We also performed a variety of statistical analyses. We used a Spearman's correlation analysis to test the relationship between the number of mentioned plants/uses and informants' age. We performed a Mann-Whitney test to compare the distribution of TABs knowledge between male and female informants and to test differences between UV values calculated for different preparations. We also performed a multivariate analysis to establish similarities and differences in ingredient composition between recipes recorded in the Alta Valle del Reno and those reported in bibliographic sources. Data were entered into a database in the form of presence/absence (1/0 in the cell) of a given ingredient in a given recipe. The final crude matrix consisted of eight units—which are the ingredients lists from different sources—and 92 variables, which are the ingredients (i.e., specific plant parts) belonging to 76 plant species. In order to limit the risk of over-fitting due to a small dataset with many attributes, a Principal Coordinates Analysis (PCoA) was used to reduce the data set dimensionality to eight coordinates, explaining 100 % of the variability. A cluster analysis was then carried out on PCoA scores, using a Ward’s cluster grouping (minimum variance method), in order to minimize the square sum inside those groups, i.e., the errors square sum [68]. Additionally, we used a Sørensen's similarity index. We also calculated the same index on ingredient usage among different sources.

## Results and discussion

### Beverages and beverage preparations

During the field survey, we collected information about all four types of alcoholic beverages, with a total of 222 citations. Specifically, the most cited TABs belong to the category of liquors (34 species, 130 citations, 43 informants), followed by fermented beverages (14, 66, 32), aromatized wines (11, 18, 8) and distillates (6, 8, 7). The same order was found when ranking types of beverages according to the adopted Use Value index (*UV*_general_): liquors 2.06, fermented beverages 1.05, aromatized wines 0.29 and distillates 0.13. When differentiating between current and past uses (i.e., uses not practiced anymore by the informant), homemade production of traditional liquors proved to be still quite common in the studied area (*UV*_current_ = 1.46; *UV*_past_ = 0.60; Mann-Whitney test: Z = 3.17; *P* < 0.01, while the use of fermented beverages mostly exists as a cultural record, surviving only in the memory of local people (*UV*_current_ = 0.24; *UV*_past_ = 0.81; Mann-Whitney test: Z = -2.98; *P* < 0.05). Differences between *UV*_current_ and *UV*_past_ were not statistically significant for both aromatized wines and distillates. Most informants reported producing/using (or have produced/used) TABs personally (total *UV*_effective_ = 2.95; total *UV*_potential_ = 0.56). This is especially true for liquors (*UV*_effective_ = 1.71; *UV*_potentia_l = 0.35; Mann-Whitney test: Z = 5.30; *P* < 0.001) and fermented beverages (*UV*_effective_ = 0.92; *UV*_potentia_l = 0.12; Mann-Whitney test: Z = 3.39; *P* < 0.001).

Our findings suggest the importance of liquors in the local communities, as a common and vivid tradition. This is probably because, compared to other types of TABs (namely distillates), liquors are relatively easy to make, have a long shelf life and are still perceived as tasty and enjoyable. Fermented beverages and aromatized wines, also very easy to prepare, are considered by local people as outdated or “out of fashion,” probably because they do not meet the local consumers’ taste requirements any longer.

Most liquors are prepared via simple manufacturing processes, the main steps being flavoring, sweetening and maturation. In many cases, informants reported the production of liquors and aromatized wines by a simple mixing of components. Personally collected plant ingredients are mixed with bought ones, namely exotic spices, sugar, alcohol/spirits or wine (the latter is homemade in some cases). The extraction of flavoring is usually carried out by maceration of raw, whole or chopped leaves (and less frequently flowers, fruits or other parts). Fermented beverages are obtained from different kinds of wild and cultivated fruits, as fermentation is also considered a cheap and energy-efficient way of conserving them. Fruits are put in glass jars and covered with sugar, then the jars are tightly closed and exposed to sunlight for a variable period of time (from few days to few months). In this way, both preserved fruit and a fermented juice are obtained, and the juice can be drunk separately, either in pure form or diluted with water. Distillation in the past was a community process at the village level. A single alembic was shared by the whole community, with the purpose of distilling “grappa” from fermented grapes or other fruit juices. “Grappa” was drunk as a spirit or used to prepare aromatized liquors. For this last practice, it was replaced by the use of 95 % alcohol as soon as it became available in the valley (approximatively around the 1950s). In Appendix 1, we provide a list of preparations and recipes drawn from the field interviews.

### Plants

**Fig. 2:**
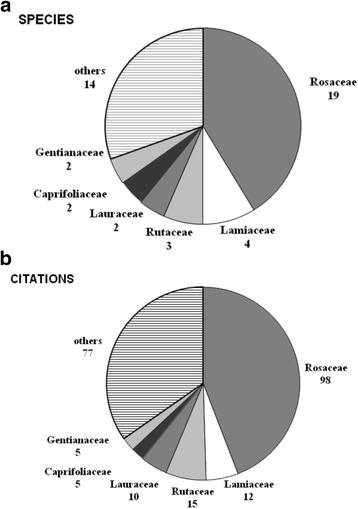
Botanical families of Traditional Alcoholic Beverages (TABs) in the Alta Valle del Reno: **a** number of species; **b** number of citations

According to our survey, 46 plant species, belonging to 32 genera and 20 botanical families, are used to prepare homemade TABs (Table 1). In the survey carried out in the Alta Valle del Reno on all traditional plant uses, 259 species were mentioned (Egea, in prep.); this means that a relevant fraction (17.8 %) of the plants of ethnobotanical interest is used to prepare alcoholic beverages. Rosaceae was the most significant family used to prepare TABs, with the largest number of both citations and cited species (98 and 19, respectively), followed by Rutaceae (15 and 3) and Lamiaceae (12 and 4) (Fig. 2). Two species for each family were mentioned for Lauraceae, Caprifoliaceae and Gentianaceae, while the remaining 14 families were represented by only one species. These results indicate a clear prevalence of fruit plants that are both cultivated (Rosaceae, Rutaceae) and wild (Rosaceae), the latter including several wild forest plants that can be regarded as Non-Wood Forest Products, according to the definition of FAO [69]. Yet, aromatic plants (Lamiaceae, Rutaceae) are also important. Sõukand et al. [27] also found Rosaceae to be the most relevant botanical family in the preparation of fermented drinks in East Europe, followed by Poaceae. This is likely related to the high content of simple and complex carbohydrates in fruit/seeds of plants belonging to these families. As noted by Sõukand et al. [27], many plants belonging to the Rosaceae family are also rich in phenols, a group of substances playing an important role as anti-oxidants in the human diet [70]. Fermented drinks made with Rosaceae—but also with other polyphenolic-rich fruits (e.g., walnuts, *Juglans regia*) —may have played some role as “detoxifying foods” in winter, in such regions where fresh vegetables are scarcely available in cold months and diet has been based for centuries on carbohydrates and saturated fats (but see below some further considerations on nutraceutical value of TABs).

Most of the ethnobotanical formulations of TABs (164 citations, 73.9 %) were extremely simple, involving just one species (38 species, 82.6 % of the total). Nineteen species (41.3 %) were used in combination with others; among these, lemon (*Citrus limon*), cloves (*Syzygium aromaticum*) and cinnamon (*Cinnamomum verum*) were the most popular and cited species used for aromatizing.

According to the general Use Value index (UVgeneral), the most important plant used for TABs (Table 1) is sour cherry (*Prunus cerasus*) (UVgeneral = 0.44). This is followed by other cultivated and wild fruit plants like walnut (*Juglans regia*) (0.38), raspberry (*Rubus idaeus*) (0.27), blackthorn (*Prunus spinosa*) (0.22), lemon (0.21), blueberry (*Vaccinium myrtillus*) (0.19) and cornel (*Cornus mas*) (0.16), and by aromatic plants such as cloves (0.16), cinnamon (0.13) and juniper (*Juniperus communis*) (0.11). With 23 informants and 24 citations (36.5 and 10.8 % of the total, respectively), walnut was found to be the most quoted species. Most of the plants with high *UV*_general_ values are mentioned as important components of TABs in other ethnobotanical studies [63, 71]. Sõukand et al. [27] report sour cherry and blackthorn as the most common liquoristic species in East Europe, followed by blueberry and juniper. When taking into consideration current and past uses, species with the highest *UV*_current_ values were *Juglans regia* (0.254), *Prunus cerasus* (0.238) and *Citrus limon* (0.159). Thirteen species were reported by the informants as used only in the past. Species with the highest *UV*_past_ values were *Rubus idaeus* (0.222), *Prunus cerasus* (0.206) and *Prunus spinosa* (0.143). These findings highlight the high difference existing between “knowledge” and “use”: some species, like *Rubus idaeus* and *Prunus spinosa*, well-known in the local alcoholic beverage tradition (relatively high *UV*_general_ values), were mainly used in the past but are infrequently used now (relatively high UV_past_ and low *UV*_current_ values). In other cases, some uses of a given species (e.g., *P. cerasus*) are still practiced (relatively high *UV*_current_ values), while others remain alive only in the informants’ memory (relatively high *UV*_general_ and *UV*_past_ values). Most informants reported a personal use of the recorded species, with few exceptions (*Castanea sativa* and *Sambucus ebulus*, whose fruits were used in the past to prepare distillates). The highest *UV*_effective_ values were obtained by *Prunus cerasus* (0.413), *Juglans regia* (0.254), *Rubus idaeus* (0.222) and *Citrus limon* (0.206), revealing the personal experience of most informants in preparing alcoholic drinks with these species.

**Fig. 3:**
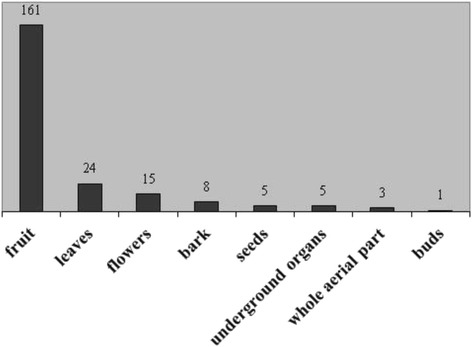
Plant parts used for Traditional alcoholic Beverages (TABs) in the Alta Valle del Reno Plant parts (X-axis). Number of citations for each used part (Y-axis)

Two plants are used in three different categories of beverages, thus resulting in the most versatile species: sour cherry (fermented beverages, aromatized wines and liquors) and blackthorn (distillates, aromatized wines and liquors). Plant parts most commonly used to prepare TABs are fruits, followed by leaves and flowers (Fig. 3). According to Pignatti [59], more than a half of the species mentioned by local informants for preparing TABs grow wild (or are both wild and cultivated) in at least one of the two regions considered in this study (28 species, corresponding to 60.9 %). Out of these, 21 (that is, 45.7 % of the total) are rather common in Italy and 7 (15.2 %) are rather rare (in a scale from “extremely common” to “very rare” adopted in Pignatti, [59]). Yet, according to the informants’ perception, some of the plants used to prepare TABs (e.g., *Gentiana* cfr. *asclepiadea*, *Vaccinium myrtillus*, *Juniperus communis*, *Fragaria vesca*, *Rubus idaeus*, *Sorbus domestica* and *Matricaria chamomilla*) are less common today than in past times or have even disappeared due to different factors. Among these factors, the informants reported changes that occurred in traditional land use and resulting effects on environmental and floristic diversity, as well as proliferation of wild ungulates in recent years. Out of the species used for TABs, only *Vaccinium myrtillus* is included among the plants considered rare or deserving of any phytogeographic interest in the floristic report concerning the regional park of Suviana and Brasimone lakes [72]. According to regional laws on biodiversity, *Gentiana asclepiadea* and *Gentianopsis ciliata* are protected in Emilia-Romagna (LR 2/77), while no species recorded for TABs is protected in Tuscany (LR 56 2000).

**Fig. 4:**
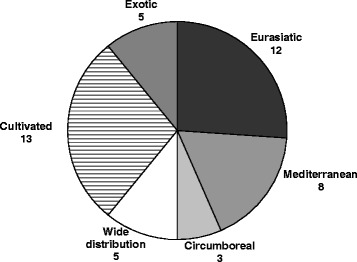
Chorologic spectrum of species used for Traditional Alcoholic Beverages (TABs) in in the Alta Valle del Reno, showing their geographical distribution (chorologic types)

General geographical distribution (chorology) of the species (Fig. 4) highlights the importance in the preparation of TABs of cultivated and exotic plants and of widely distributed species (including adventitious ones). Mediterranean species play only a very marginal role as ingredients of TABs in the studied area.

**Fig. 5:**
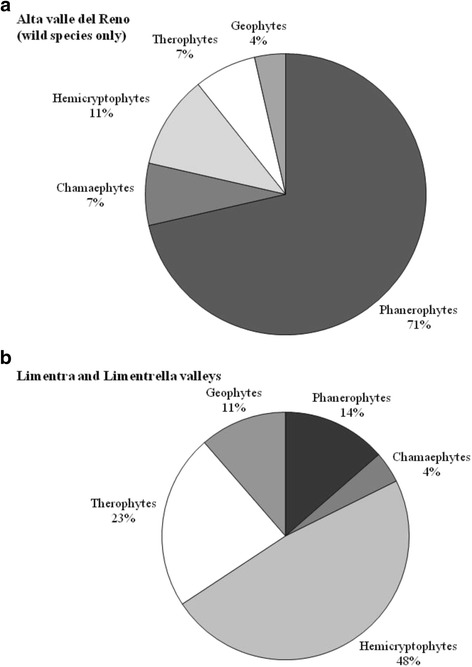
**a** Biological spectrum of species used for Traditional Alcoholic Beverages (TABs) in in the Alta Valle del Reno. **b** Biological spectrum of species of the Flora of Limentra Orientale and Limentrella valleys (data from Venturi [55]). Phanerophytes: trees and shrubs with perennating buds borne more than 25 cm above the ground; Chamaephytes: small shrubs with buds close to the ground; Hemicryptophytes: perennial herbs; Geophytes: perennial herbs with underground stems; Therophytes: annual herbs

The biological spectrum (i.e., the subdivision of species into different life-forms) shows that plants used to make alcoholic beverages in the Alta Valle del Reno are mostly Phanerophytes (76.1 %), that is, woody plants (27 trees, eight shrubs). The prevalence of Phanerophytes is also confirmed when only wild species (28) are considered (see Fig. 5). Such a biological spectrum would be quite abnormal if referred to the flora of any area lying in North-Central Italy in similar sub-mountain or mountain environments: at such latitudes and altitudes, perennial herbs (Hemicryptophytes) are always the predominant life form, as is confirmed by the biological spectrum drawn from the flora of Limentra Orientale and Limentrella valleys (provinces of Pistoia and Prato) [73], a territory also including a part of the area investigated in the present study. We must stress that in biological spectra, only the presence or absence of a species is considered, regardless of its abundance. This is the reason why in regions characterized by cool temperate phyto-climates hemicryptophytes (i.e., perennial herbaceous plants) are commonly the prevalent life form, even in areas dominated by woody plants communities. Accordingly, such climates are commonly known as “hemicryptophytic climates” (see among the others Packham et al. [74]). This means that plants collected for TABs in the study area are not evenly distributed in the local flora: on the contrary, people choose only a few species that share some peculiar features, namely fruit with high sugar content (wild and cultivated fruit trees/shrubs) and/or different plant parts with strong aromatic flavors (mostly aromatic shrubs). Herbs, which have a fundamental ethnobotanical importance in Italian tradition as wild food in the form of wild salads and vegetables [64, 75], are almost of no interest as a source of homemade alcoholic drinks.

### Informants and socio-cultural context

In the studied area, homemade TABs have an important traditional and cultural value. Among the 113 informants interviewed during the whole ethnobotanical research carried out in the Alta Valle del Reno (Egea, in prep.), 63 (55.7 %) mentioned plant-based TABs: 21 men and 42 women (Table 2). Women appear to hold a wider knowledge of TABs than men: they reported the use of a higher number of species (L_s_), a higher number of uses (i.e., citations, L_u_) and also more details about beverage preparation. Moreover, all the informants who mentioned more than five species used for making TABs were women. Although all these differences were not statistically significant, these findings suggest that women play a key role in maintaining the traditional knowledge and related skills for the preparation of alcoholic beverages in the Alta Valle del Reno. On the other hand, when considering all the uses mentioned by the informants in the whole ethnobotanical research, women proved to be significantly more knowledgeable than men, as showed by non-parametric analysis (Mann-Whitney U test). Both the ratio L_s_/T_s_ (number of species used for TABs/number of species cited for any ethnobotanical use by each informant) and the ratio L_u_/T_u_ (number of TAB uses/number of any ethnobotanical use) were statistically significant (Z = 2.56, *P* < 0.05; Z = 2.62, *P* < 0.01, respectively). This means that, within their whole ethnobotanical knowledge, women hold a wider competence concerning TABs than men.

Over 90 % of the 63 informants reporting the use of plants for TABs were over 60 years old (58 informants); of these, 39 (61.9 % of the informants quoting plant-based TABs) were aged between 61 and 80. Meanwhile, 19 (30.2 %) of the informants were over 80. Only five (7.9 %) informants were younger than 60, more precisely, between 43 and 59 (Table 2). Spearman’s correlation analysis showed significant negative differences between the age of the informants and the number of both mentioned species (r = -0.26, *P* < 0.05) and different uses (r = -0.25, *P* < 0.05), indicating that the higher the age of the informant, the lower the knowledge of TABs and vice versa. Yet, it must be underlined that almost all the informants were quite elderly people anyway. As for education level, most of the informants (36, i.e., 57.2 %) received only some primary education (“scuola elementare”), 15 (23.8 %) had attended middle school (“scuola media”), six (9.5 %) high school, and six held a university degree. Education level of informants proved not to be significantly correlated with any of the considered variables. These findings appear not to be in accordance with other ethnobotanical studies, whose results show that knowledge of both plants and plant uses generally increases with age and decreases with education level, at least in those communities suffering a strong erosion of traditional knowledge ([76, 77] also for further references on this topic). Our data suggest instead that knowledge on TABs, owned mainly by elderly people of the community in the past, are very popular across different age and education groups, suggesting a possible transmission still ongoing across generations and social classes. These data are consistent with considerations reported by Sõukand et al. [27], who affirm that traditional drinks are currently revalued even among relatively young people, as they provide unusual flavors and other peculiar taste characteristics, in this way increasing the consumer’s experience of food.

Data reported in Table 3 clearly highlight the cultural importance of TABs in the Alta Valle del Reno, compared to other Italian areas of different extensions: the whole of Italy [71], Tuscany [63], and the municipality of Firenzuola, in the neighboring the Alta Valle del Sieve [65]. Although they are drawn from surveys carried out by different researchers and with different focuses, data can be legitimately compared, as they appear to be based on the same basic concept of ethnobotanical use: i.e., cultivated plants are considered of ethnobotanical interest only when the traditional (ethnobotanical) use is not the main use for which these plants are commonly grown [66]. In comparing data, we only excluded from our dataset three exotic spices (cinnamon, cloves and vanilla) recorded during our field investigation and not considered in the other datasets, as these three plants are cultivated elsewhere and only purchased in stores in the study area. The ratio between the number of species used for TABs and the total number of species mentioned for any ehtnobotanical use (L_s_/T_s_) is almost three times higher for the Alta Valle del Reno than for the whole of Italy, and the same was found for the other considered areas. Similarly, the ratio between the number of species used for TABs and the total number of species mentioned for any food use (L_s_/F_s_) in the Alta Valle del Reno reaches twice the values calculated for the other considered areas. These results show that TABs play an important role in the traditional gastronomy of the Alta Valle del Reno compared with other parts of Italy. Considering traditional alcoholic beverages, this must be regarded as a relevant component of traditional knowledge of plant uses of this area and as a typical trait of the way people live and socialize in local communities.

Drinking wine and other alcoholic beverages has been a typical cultural feature of this mountain culture since the 13^th^ century, when taverns were managed by the Sambuca Statutes (1291-1340) [55]. As it is typical of most sub-mountainous villages in the Mediterranean area, in the Alta Valle del Reno taverns have always been places where men used to drink and buy wine, relax, socialize and have fun together. Informants affirm that drinking wine in taverns or at home was once perceived as almost the only available source of entertainment.

Preparation of homemade alcoholic drinks requires efforts and time, and ingredients are sometimes hard to collect. Consequently, TABs are perceived as something special and somehow precious. Moreover, recipes are often perceived as a heritage and so acquire a very special meaning. Ethnobotanical knowledge is usually passed on from generation to generation through oral transmission, but in the studied communities many informants held written recipes of TABs, left by their mothers, grandmothers, or other members of the family. During some interviews, a kind of jealousy about familiar recipes was perceived: most informants were willing to share the general way of preparation, but not the exact dosages or similar specific details.

Traditional homemade alcoholic drinks are prepared to be shared and offered to friends and special guests, especially during cold winter time. Such beverages are never exchanged for money, as they are intended as symbols of hospitality and gratitude. They are even offered for free by restaurant owners to customers after meals. TABs act as a sort of “social lubricant,” giving special importance to meals, celebrations and other social occasions.

### Cultural influence of Tuscany and Emilia-Romagna

**Fig. 6:**
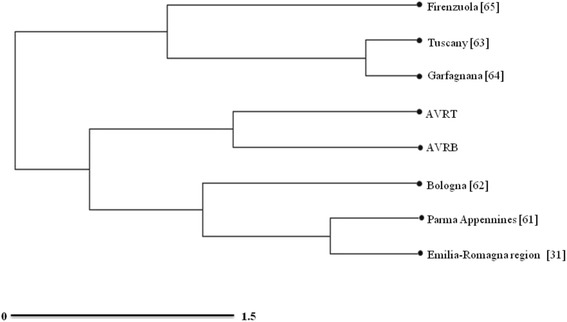
Hierarchical tree representing the relationships between the different analyzed sources based on the frequency of ingredients, according to Ward’s minimum variance criterion

We analyzed the influence of different cultures from Tuscany and Emilia-Romagna on the preparation of TABs using statistical multivariate analyses. A total of eight data sources were compared, consisting of plant ingredients of TABs in different areas. Even though data were drawn from studies carried out by different authors and with different methods and focuses, the results of cluster analysis are nevertheless worthy of interest. As shown in Fig. 6, three main groups were pointed out. The first group includes all the sources from the region Emilia-Romagna: the whole Emilia-Romagna region [31], Bologna [62], and Parma Apennines [61]. In the second cluster, closely related to the previous one, data collected from the two sides of the Alta Valle del Reno lie very closely to each other, revealing that knowledge is quite homogeneously distributed in the whole study area. The third cluster is formed by sources from Tuscany: the whole of Tuscany [63], Garfagnana [64] and Firenzuola, [65]. The fact that the Alta Valle del Reno clusters more closely with sources from Emilia-Romagna than with the ones from Tuscany suggests that TAB knowledge in the study area is more affected by the Emilia-Romagna liquoristic tradition. These results were also confirmed by those obtained using the Sørensen similarity index, comparing the same eight sources (results not reported).

### Nutraceutical and ethnomedicinal aspects

In the studied area, TABs appear not only to be part of the traditional food culture, but also to belong to the local traditional medicine. This is different from what was reported in Sõukand et al. [27] for East Europe, where only a few of the recorded fermented products are perceived by local population as “folk nutraceuticals” (i.e., plant products taken as food in order to maintain a status of health, according to Pieroni and Quave [78]); in the Alta Valle del Reno, a considerable number of species (32, i.e., 70 % of all the species locally used to prepare TABs) are identified by the informants also as medicinal plants traditionally used to heal or prevent different kinds of disease, under different forms of preparation and administration. Out of these, for 16 species (i.e., 34.8 % of all the species used for TABs), the medicinal remedy is represented by the alcoholic beverage itself, which can be drunk either as a recreational beverage or as a medicine. Most of these alcoholic preparations are used in treating or preventing digestive system diseases, especially dyspepsia (13 species, i.e., 81.3 % of species used to prepare medicinal TABs), followed by remedies against cough and other respiratory system diseases (two species, 12.5 %) and by treatments used for high blood pressure or as a general preventive measure (one species each, 6.3 %). The main alcoholic preparations used also as medicinal remedies are liquors (13 species, 81.3 % of all those used for TABs), followed by aromatized wines (four species, 25 %) and fermented beverages (three species, 18.8 %) [2]. The role of homemade spirits as food medicine has been highlighted also by Pieroni [64] in a study carried out in the upper Lucca province (North-Western Tuscany).

Maceration of herbs and spices in wine or alcohol to obtain medicinal alcoholic beverages has been a common practice since antiquity. The invention of aromatized wines is attributed to Hippocrates (5^th^- 4^th^ century BC), the Greek physician considered the father of western medicine. Some studies suggest that the “Hippocratic wine,” commonly used in ancient Greece as a digestive, was produced by macerating dittany (*Origanum dictamnus* L.) and wormwood (*Artemisia absinthium* L.) flowers. More recently, vermouths (whose origins date back to 17^th^ century) represent a special case of aromatized wines, prepared with complex mixtures of aromatic plants that have been considered to be medicinal remedies for a long time. The use of complex mixtures of plants in medicinal beverages (non-alcoholic in this case) is still common in Asia [79]. In addition to aromatized wines, liquors have a centuries-old history, and some of them find their origin in traditional pharmacopoeia. Some examples are the French liquors *Chartreuse* (known from the beginning of 17^th^ century as an “elixir of long life,” containing 130 different botanicals combined in an alcohol base) and *Benedectine* (whose original recipe goes back to the Renaissance and contains 27 different botanicals) [28]. Aromatized wines, still widely known in different cultures for their “warming” and “strengthening” qualities, are especially typical of mountain zones and are also locally reported as medicinal remedies to treat respiratory ailments. According to our results, in the Alta Valle del Reno hot aromatized wines (“*vin brulé*,” i.e., mulled wine) made with wine and aromatic plants and/or spices are prepared and offered mainly in winter to cope with cold weather.

For their high vitamin and sugar content, fruit-based fermented beverages are perceived by most informants in the studied area both as recreational drinks and as a good support for general body health. In summer, these beverages—sometimes mixed with water—are commonly consumed as refreshing drinks. On the other hand, according to some informants, in the past they were considered important in winter to integrate the poor seasonal diet, mainly based on chestnut flour. Actually, many underutilized wild fruits used for TABs such as *Rosa canina*, *Sambucus nigra*, *Vaccinium myrtillus*, *Rubus ulmifolius*, *Prunus spinosa*, *Prunus cerasus* and others have great potential as functional foods, providing chemical compounds with nutraceutical and medicinal properties [80–84]. To verify the supposed medicinal and nutraceutical properties of the alcoholic beverages recorded in this study, it will be necessary to evaluate the levels of these compounds.

## Conclusions

The results of this study point out that TABs occupy an important place in the traditional culture and social life of this area. This is shown by the variety of species used, the high percentage of informants citing TABs preparations (over half the informants who cited any ethnobotanical use), and the high number of citations. Although some of the reported uses merely survive in the informants’ memory, many of them are still current, despite the deep socio-ecological transformations that have characterized human settings in this area during the last 50 years, and are still appreciated and practiced by people of different ages, not only by the elderly as is commonly the case with traditional ethnobotanical knowledge. Local specificity and richness of this kind of traditional culture show up clearly when comparing field data on TABs recorded in the Alta Valle del Reno, with those drawn from different sources concerning Tuscany and the whole of Italy. This richness is perhaps related to the peculiar position of the area—a biogeographical, linguistic, cultural, and historical borderland—and with its relative geographical isolation, which contributed to maintaining a strong cultural identity in local communities.

Retrieving local knowledge of TABs could be regarded as a means not only of contributing to the conservation of local traditional knowledge, but also of reevaluating a possible source of endogenous rural development. TABs’ small-scale production could supply local shops and restaurants that will benefit from gastronomic tourism, attracting visitors’ interested in local traditional foods connected with local natural resources. Nutraceutical or even therapeutic properties of some of the plant ingredients used for TABs and well-known by local people could enhance the value of these products. Yet, as made clear in the interviews, in the studied area TABs are not merely considered as foods or medicines: they used to be—and still are—a peculiar trait of local culture, a link with past generations and a part of the local way of living together. To share a glass of homemade liquor with friends and guests after dinner, especially during the hard winter time, is primarily a way to feel a part of the same community and to share the same history and environment.

## Abbreviations

AVRB, Alta Valle del Reno, Emilian side; AVRT, Alta Valle del Reno, Tuscan side; TABs, Traditional alcoholic beverages; UV, Use Value Index

## Appendix 1

**Table 4:** Traditional Alcoholic Beverages (TAB) in upper Reno valley (AVR). Plants, preparations, recipes

Plant	Recipe
*Aloysia citriodora* Palau	- Lemon verbena liquor. Steep 67 *Lippia* leaves and lemon zest with 500 g sugar in 800 ml water and 500 ml alcohol for 21 days. Shake daily.
*Camellia sinensis* (L.) Kuntze	- Granny's ‘Cent'erbe’ (100 herbs liquor) . Steep 5- 6 sage leaves, 5- 6 basil leaves, one teabag, pine buds, rosemary and wild mint in alcohol for 21 days, then filter and add a syrup made with 100 g sugar boiled in 100 g water. - ‘Cento erbe’. Steep 3 laurel leaves, 3 lemon leaves, 3 juniper ‘berries’, 3 chamomile ‘flowers’, 3 tea leaves, 3 sage leaves, 3 rosemary leaves in a mixture of 350 g sugar boiled in 1 l water (then cooled) and 40 g 90 % alcohol.
*Castanea sativa* Miller	- Chestnuts are used to make a distillate (‘grappa’).
*Cinnamomum verum* J. Presl	Used for flavoring different liquors: - ‘Nocino’ (see *Juglans regia*) - ‘Corniolino’ (see *Cornus mas*) - Juniper liquor (see *Juniperus communis*) - ‘Vin brulé’ (mulled wine), prepared in winter to drink during the ‘veglie’ (evenings spent at home with friends). Heat the wine to boiling with lemon/orange zest (or with an apple quarter), cinnamon and cloves. Alternative: when boiling, set fire to the wine to remove alcohol.
*Citrus limon* (L.) Burm. f.	- ‘Limoncino’. Steep 3-4 lemons in 1 l alcohol. Filter. Make a syrup with 1 kg sugar boiled in 1 l water, let cool and mix with the infusion . - Lemon liquor. Steep 10 lemon leaves in alcohol. Used also for flavoring different beverages: - ‘Cento erbe’ (see *Camellia sinensis*) - ‘Nocino’ (see *Juglans regia*) - ‘Corniolino’ (see *Cornus mas*) - Lemon verbena liquor (see *Lippia triphylla*) - ‘Vin brulé’ (see *Cinnamomum verum*).
*Citrus sinensis* (L.) Osbeck	- Used for flavoring ‘Vin brulé’ (see *Cinnamomum verum*).
*Coffea arabica* L.	- Used for flavoring in an alternative receipt of ‘Nocino’ (see *Juglans regia*).
*Cornus mas* L.	- ‘Corniolino’. Steep 1 kg of *Cornus mas* (cornelian cherry) fruits, 5 cloves, 1 pinch cinnamon and the zest of 1 lemon in 1 l alcohol for 40 days, then filter. Make a syrup with 625 g sugar boiled in 625 ml water, let cool and mix with infusion. Alternative: syrup made with 400 g sugar in 1 l water. - ‘Corniole’ liquor. Made with water, alcohol, sugar and *Cornus mas* fruits. - Steep *Cornus mas* fruits in grappa for 1 month, then filter. - Put 1 kg *Cornus mas* fruits and 750 g sugar in glass jar, pour enough red wine to cover. Steep for 2-3 months, then filter. Add 300 g alcohol.
*Fragaria vesca* L.	- Fill glass jar with wild strawberries, cover with sugar, close jar and expose to sunlight for 40 days. The lightly fermented syrup, filtered and bottled, is used as a drink, diluted with a double quantity of water.
*Gentiana* cfr. *asclepiadea* L.	- ‘Criptamarol’ liquor. Prepared with alcohol and gentian roots by a priest (expert in botany) who offered the liquor to guests or used it for barter; nobody else knew the recipe. - Gentian liquor (digestive, remedy for gastritis). Roots collected from wild plants, cleaned and steeped in grappa. - Gentian-aromatized wine (digestive, remedy for gastritis). Roots collected from wild plants, cleaned and steeped in wine.
*Gentianopsis ciliata* (L.) Ma. (cfr.)	- Gentian liquor (see *Gentiana* cfr. *asclepiadea* L.) - Gentian-aromatized wine (see *Gentiana* cfr. *asclepiadea* L.)
*Juglans regia* L.	- ‘Nocino’. The most popular traditional liquor in the area, usually offered as a digestive after meals, to warm up in cold weather, as a drink to visiting friends. According to some informants, the macerated fruits remaining after filtering are washed in alcohol to make a lighter liquor, drank also during snacks. The preparation of ‘Nocino’ often requires precise rituals: gathering of walnuts on Midsummer Day, ‘magical’ number of fruits, cross-shaped cutting of the fruits before preparation. According to one informant, the nuts should be stolen, walking barefoot. Walnuts are gathered unripe, when their hulls are still green. Spices and flavorings are often added. The liquor must ‘stand’ a long time before consumption. Different recipes: . Steep 30 nuts, 750 g sugar, 2 g powdered cinnamon, 10 cloves and lemon zest in 1.5 l alcohol and 4 l water for 40 days in the dark, then filter. . Steep 21/22 nuts, 500 g sugar, 1.5 g cinnamon and 7 cloves in 1 l alcohol and 0.5 l water for 3 weeks, then filter. . Steep 19 nuts (broken in half) in 1 l 90 % alcohol for 40 days, then filter and add a syrup made with 400 g sugar boiled in 800 ml water. Let stand for 1 month before consuming. . Steep 33 nuts (pierced with a fork and cut into quarters) and 750 g sugar in 1.5 l alcohol and 0.5 l water. Use glass jar big enough to be half filled by the mixture. Close jar and expose to sunlight for 60 days, shaking it every 4-5 days, then filter. . Steep 28 nuts (broken in half), 900g sugar (dissolved in hot water), the zest of 1 lemon, 1 vanilla bean, 4 cloves, 1 pinch cinnamon in 1 l 95 % alcohol for 40 days. . Steep 1 kg nuts and 1 kg sugar in 1 l alcohol and 1 l water for at least 1 year (or 5-6 years; the longer the better), then filter. . Steep 2 kg of nuts (broken in small pieces) in 1 l alcohol, in the dark, for 7-8 days. The jar must be airtight. Filter, then add a syrup made with 1 kg sugar in 1 l water. Bottle and let stand (the longer the better). . Steep 15 nuts (cut into quarters), 0.5 kg sugar, juice and zest of 1 lemon, 1 tbsp coffee in 0.5 l water and 0.5 l 90 % alcohol in glass jar. Expose to sunlight for one day, shake to dissolve sugar, let stand for 40 days, filter and bottle. . Steep 25 nuts with sugar in 1 l alcohol for 40 days, then filter. . Steep nuts with sugar, lemon and cloves in wine and alcohol (quantities are kept secret) for 40 days, exposing to sunlight. Filter and bottle. - ‘Nociato’. Take same nuts and same jar used to prepare ‘Nocino’. Cover fruits with vermouth or dry white wine, let stand for 1 week, filter. Keep in a cool place and serve as aperitif.
*Juniperus communis* L.	- Juniper liquor (digestive). Different recipes: . Steep 1 small branch of juniper with ‘berries' in grappa for 40 days. . Steep 1 ‘giumella' (handful) of unripe and 1 of ripe juniper ‘berries', some cinnamon and 3 cloves in 1 l 90 % alcohol and a syrup made from 1 kg sugar dissolved in 1 l water. Keep in airtight jar for at least 40 days. Filter and bottle. . Steep juniper ‘berries' and sugar in alcohol or grappa. - ‘Cento erbe' (see *Camellia sinensis*). - The ‘berries' are also used to make grappa.
*Laurus nobilis* L.	- Laurel liquor. Steep 20 laurel leaves with 600 g sugar in 700 g water and 1 l alcohol for 3 days. Boil for 5 minutes, let cool, filter and bottle. - ‘Cento erbe' (see *Camellia sinensis*).
*Malus domestica* Borkh.	- Apple seeds liquor. Steep 1 glassful of apple seeds (old cultivar) in 300 g 95 % alcohol for 10 days in closed jar. Shake jar daily. Make a syrup dissolving 400 g sugar in 400 ml water, heat until boiling then let cool. Add syrup to infusion and let stand for 40 days. - Alternative version of ‘vin brulé’ (see *Cinnamomum verum*).
*Malus sylvestris* Miller	- The same as *Malus domestica*.
*Matricaria chamomilla* L.	- ‘Cento erbe' (see *Camellia sinensis*).
*Mentha spicata* L. (s.l.)	- Granny's ‘Cent'erbe’ (see *Camellia sinensis*). - ‘Mentarello’ liquor.
*Ocimum basilicum* L.	- Granny's ‘Cent'erbe’ (see *Camellia sinensis*). - Basil liquor (digestive). Steep a handful of basil leaves with 1 kg sugar and the zest of 1 lemon in 1 l alcohol for 40 days, then filter. Alternative: steep 150 basil leaves in 1 l alcohol for 3 days, then filter, add syrup made with 1 kg sugar in 1 l of water, bottle.
*Pinus nigra* J.F. Arnold (s.l.)	- Granny's ‘Cent'erbe’ (see *Camellia sinensis*).
*Prunus avium* L.	- Fill jar with pitted cherries, cover with sugar, close and expose to sunlight for 40 days. The result is preserved cherries and a fermented juice that can be used separately as a drink. - Cherries in alcohol. Steep fruits (preferably the ‘duroni’ variety) in alcohol in glass jar, let stand. The result is preserved cherries and a cherry-flavored alcohol. - Cherry liquor. Steep 1 kg cherries with 1 kg sugar in 1 l alcohol and 1 l water for 1 year (better 5-6 years). Filter and bottle, or leave the cherries in the liquor to eat.
*Prunus cerasifera* Ehrh.	- ‘Wild plums’ are used to make grappa.
*Prunus cerasus* L.	- ‘Marasche’ in sugar. Cut the fruit stalks leaving a 0.5 cm piece attached (alternative: half fruits with a piece of stalk left, half without). Fill jar with fruits and cover with sugar (1 kg fruits with 200 g, or 7-800 g, or 1 kg sugar). Expose to sunlight (alternative: close jar and turn upside down) until sugar is dissolved (for 20, 30, 40, 60 days or the whole summer). The result is preserved fruits in a more or less alcoholic juice, called 'Maraschino' or ‘Maraschino al sole’, that can be used as a drink (pure or diluted). - ‘Marasche’ or ‘Amarene’ syrup. Press fruits and brew them for 48 hours, filter. Add 1 kg sugar for each l juice, boil for 2-3 minutes, let cool then bottle. Alternative: macerate fruits with sugar for some days, then filter and bottle. The result is a lightly alcoholic syrup, to be diluted with water and used as a drink. - ‘Cherry’. Let steep 35 leaves in 1 l red wine for 4 days, then filter. Make a syrup with 400 g sugar boiled in 400 g water, let cool, mix with 200 g 90 % alcohol and add to infusion. Let stand for 1 month. - Put fruits (leaving a piece of each stalk) in glass jar, add 4 tbsp sugar and cover with grappa. Filter after 40 days. - ‘Marasche’ liquor. Macerate pulp of 1 kg fruits and 600 g sugar for 24 hours in glass jar. Boil for 8 minutes, let stand, add 200 g of alcohol. - ‘Marasche’ pits liquor. Steep pits from 1 kg fruits in 300 g alcohol for 40 days (alternative: 40 g pits in alcohol). Make a syrup boiling 300 g sugar in 300 g water. Let cool, add to infusion, filter, let stand for 2 months. - Put fruits (transversely incised and complete with stalks) in glass jar, cover with alcohol, water and sugar (approx. 500 g each), expose to sunlight for 2-3 months. - ‘Maraschino con le foglie' or ‘Amarene’ syrup (lightly alcoholic beverage, usually diluted with water). Macerate 100 leaves (gathered in July/August) with 1 l red wine and sugar in closed jar exposed to sunlight, for 30 days. Alternative: 800 g sugar, boil for 20 minutes instead of exposing to sunlight.
*Prunus domestica* L.	- Plums are distilled to make grappa. - Fill jar with plums, cover with sugar, close and put on a window ledge for the whole summer. The result is preserved fruit and a lightly alcoholic juice.
*Prunus laurocerasus* L.	- ‘Laurino’ (digestive). Steep 350 g fruits in 1 l 95 % alcohol. After 15 days remove, crush and put back one fifth of the fruits. Add syrup made with 500 g sugar in 500 g water, filter then bottle.
*Prunus persica* (L.) Batsch	- Peach pits liquor. An after-meal drink, to be offered to visiting friends. Put 20-30 peach pits (washed with water) in glass jar, fill jar with 90 % alcohol, steep for 40 days, filter. Wash again pits with equal weight of water. Boil this water with equal weight of sugar to make a syrup. Let cool and mix with infusion.
*Prunus spinosa* L.	- Fruits are used to make grappa. - ‘Strozzighino’ or ‘strozzighi’ (*Prunus spinosa* fruits) liquor. Different recipes: . Steep 1 kg ripe fruits (gathered in December, when most sweet) in 1 l alcohol for 40 days. Stir with wooden spoon and filter with towel, wringing vigorously. Make a syrup boiling 500 g sugar in 500 g water, let cool, add to mixture. Filter again, then bottle. . Steep 1 kg fruits with 1 kg sugar in 1.5 l white wine and 1 l alcohol for 3 months, stirring from time to time. Filter and bottle. - Macerate fruits with grappa. - Put fruits in glass jar with some alcohol and sugar, macerate exposing to sunlight. - Mix 1 kg fruits with 750 g sugar, cover with red wine. Macerate for 2-3 months, filter, add 300 g alcohol.
*Pyrus communis* L.	- Pear wine. Macerate fruits gathered at the end of summer/beginning of fall (when dark brown) in wine vat for 4-5 days (alternative: 13 days).The result is a sparkling, sugary, cider-like beverage that should be consumed as soon as possible, preferably with roasted chestnuts.
*Pyrus pyraster* Burgsd.	- Wild pear wine. See *Pyrus communis* (but remove seeds).
*Robinia pseudoacacia* L.	- Brew flowers in water to make a sort of sparkling wine.
*Rosa canina* L. (s. l.)	- Rose liquor. Fill one quarter of a bottle with dog-rose petals, add 1-2 tbsp sugar, fill with grappa. Let stand for 1 month, then filter.
*Rosa* cv.	- ‘Rosolio’. Very sweet liquor, offered to guests in specific tiny glasses ('servito da rosolio'). Put rose petals and pounded sugar (in layers) in glass jar. Cover with towel and expose to sunlight. When sugar is dissolved, add water and alcohol, macerate for some time then filter. - Grappa flavored with leaves of an ancient rose variety.
*Rosmarinus officinalis* L.	- Granny's ‘Cent'erbe’ (see *Camellia sinensis*).
*Rubus* cv.	- Hand-squeeze fruits, macerate in pot for 8 days. Filter with towel and add an equal weight of sugar. Used as cough-medicine (diluted with water).
*Rubus hirtus* W. et K.	- See *Rubus* cv.
*Rubus idaeus* L.	- ‘Lamponata’ or raspberry syrup. Used diluted with water 1:1 o 1:2 (‘to kill the water’). Different recipes: . Wash fruits, put in bowl, squeeze by hand and brew for 20 days, stirring every morning. Filter with towel (or use vegetable mill), boil for 2-3 minutes with sugar (800 g for 1 kg mixture) and bottle. . Macerate fruits with sugar for some days (alternative: two nights), filter and bottle (alternative: boil before bottling).. . Leave fruits in bucket outside for 1 week, until mouldy (alternative: for 4-5 days, for 8 days). Filter with towel or squeeze by hand. Add 1 kg sugar for each l juice (alternative: no sugar added), put into jars (alternative: boil for 10-15 minutes before bottling), expose to sunlight for 30-40 days (alternative: boil for 30 minutes). **.** Press fruits, let stand in bucket or pot for 5-6 days (alternative: 8 days), filter with towel (or use vegetable mill). . Boil pressed fruits with some sugar, let stand until fermented. Heat juice, then filter. - Fill jar (or bottle) with fruits, cover with sugar, close jar and expose to sunlight for 40 days. The result is preserved fruit and a lightly alcoholic juice**,** used as a drink (pure or diluted with water). - Raspberry liquor. Macerate 1 kg fruits with 1 kg sugar in 1 l alcohol and 1 l water for 1 year (or 5-6 years). Filter, or leave the raspberries in the liquor to eat.
*Rubus ulmifolius* Schott	- Blackberry syrup or 'Morino', used as a drink, pure or diluted with water. Different recipes: . Macerate 1 kg fruits with 500 g sugar and some alcohol for 2 weeks. Filter to eliminate seeds. . Boil pressed fruits with some sugar, let stand until fermented. Heat juice and filter. . Leave fruits in bucket for 1 week (alternative: 4-5 days) until mouldy. Filter with towel or squeeze by hand. Add 1 kg sugar for each l juice (alternative : 1 kg sugar for each bucket), put into jars, expose to sunlight for 30-40 days (alternative: boil for 10-15 minutes then put into jars). . Let stand fruits for 2 days in a bowl until fermented, filter with hemp cloth, add 1 kg sugar for each kg juice, boil for 5 minutes. - Blackberry liquor. Macerate 1 kg fruits in 600 g alcohol for 40 days. Make a syrup boiling 600 g sugar in 500 g water, let cool and add to mixture. Filter, add strained fruits, filter again then bottle.
*Ruta graveolens* L.	- Put a small branch in a bottle and fill with grappa.
*Salvia officinalis* L.	- Granny's ‘Cent'erbe’ (see *Camellia sinensis*). - ‘Cento erbe’ (see *Camellia sinensis*). - Digestive liquor. Steep 55 leaves with 300 g sugar in 1 l grappa for 30 days, then filter.
*Sambucus ebulus* L.	- Ripe fruits, called ‘ebbi’, are used to make grappa (characterized by the unpleasant smell left by fruits during distillation).
*Sambucus nigra* L.	**-** Elder syrup, used as a drink (pure or diluted with water) and as a treatment for high blood pressure. Different recipes: . Press fruits, boil with some sugar, let stand until fermented, heat then filter. . Put ripe fruits in bucket or vat and cover with towel. Let stand for 4-5 days until mouldy. Squeeze by hand, add sugar (1 kg or 800 g sugar for each bucket). Boil juice with sugar for 10-15 minutes, bottle. Better with ageing. . Put ripe fruits in jar and let stand until fermented, press then pass through vegetable mill. - Macerate fruits in alcohol for 1 month.
*Sorbus domestica* L.	- ‘Sorbole’ wine. Macerate fruits in closed vat for 4-5 days. The result is a sparkling, sugary, cider-like beverage that should be consumed as soon as possible, preferably with roasted chestnuts.
*Syzygium aromaticum* (L.) Merr.& L.M.Perry	- Used for flavoring different liquors: . ‘Nocino’ (see *Juglans regia*). . ‘Corniolino’ (see *Cornus mas*). . Juniper liquor (see *Juniperus communis*). - Used for flavoring ‘Vin brulé’ (see *Cinnamomum verum*).
*Vaccinium myrtillus* L.	- Blueberry syrup, used as a refreshing drink, pure or diluted with water 1:2; the pure syrup is also used as a cough-medicine. Different recipes: . Press fruits, boil with some sugar, let stand until fermented. Heat the juice, then filter. . Wash fruits, squeeze by hand, put in bowl and macerate for 20 days, stirring every morning. Filter with handkerchief, boil juice with sugar (800 g for each kg juice) for 2-3 minutes, let cool and bottle. . Press fruits and let stand in bucket, covered with towel, for 5-6 days; squeeze with cloth or use vegetable mill, add sugar (500-600 g for each l of juice). Boil for a short while, then bottle (alternative: bottle without boiling and expose to sunlight for 40 days). . Put ripe fruits in bucket or vat (or pot), cover with towel, let stand for 4-5 days (alternative: 8 days) until mouldy. Squeeze by hand or filter with towel. Add equal weight of sugar (alternative: 800-1000 g sugar for each bucket). Boil for 10-15 minutes, put in jars while still hot, close and put upside down. . Boil fruits briefly in water, squeeze or pass through vegetable mill. Make a syrup boiling water with sugar, let cool and add to mixture. Filter with linen towel, bottle then boil 20 minutes to sterilize. - Fill jar with fruits, cover with sugar, close and expose to sunlight for 40 days. The result is preserved fruit and a lightly alcoholic juice. - Blueberry liquor or ‘Mirtillino’. Different recipes: . Macerate 1 kg fruits with 1 kg sugar in 1 l alcohol and 1 l water for one year (or 5-6 years, the longer the better). Filter, or leave the blueberries in the liquor to eat. . Fresh fruits mixed with equal weight of water and 3 times same weight of alcohol. . Macerate fruits in alcohol for 40 days, then filter. Make a syrup boiling sugar in water, let cool then add to mixture, stir well and bottle.
*Vanilla planifolia* Andrews	- Used for flavoring ‘Nocino’ (see *Juglans regia*).
*Vitis labrusca* L.	- Steep 1 kg of Vitis labrusca (fox grape) fruits in 1 l alcohol, 1 l water and 1 kg sugar for at least 1 year (better 5-6 years). Filter and bottle, or leave the grapes in the liquor to eat.
